# Perceived social support as a mediator of the relationship between fear of progression and post-traumatic growth in lung cancer patients undergoing chemotherapy: a structural equation model analysis

**DOI:** 10.3389/fpsyt.2026.1906123

**Published:** 2026-08-26

**Authors:** Fan Xu, Qiao Li, Jing Wang, Shaoju Xie, Jiquan Zhang

**Affiliations:** 1 Oncology Department, Deyang People’s Hospital, Deyang, Sichuan, China; 2 Nephrology Department, Deyang People’s Hospital, Deyang, Sichuan, China

**Keywords:** chemotherapy, fear of progression, lung cancer, mediation effect, perceived social support, post-traumatic growth

## Abstract

**Background:**

Post-traumatic growth (PTG), as a positive psychological adaptation mechanism, can significantly enhance patients’ psychological resilience and promote positive psychological transformation. However, among lung cancer patients undergoing chemotherapy, fear of progression (FoP) is associated with substantial psychological distress and lower levels of positive adaptation. Research on the association between FoP and PTG in this population remains limited.

**Objective:**

To investigate the association between FoP and PTG among lung cancer patients undergoing chemotherapy, and to examine whether perceived social support (PSS) statistically mediated this association.

**Methods:**

A cross-sectional design was conducted among lung cancer patients undergoing chemotherapy at a Grade A tertiary hospital in Deyang City from March to July 2025. Data collection involved the use of a general information questionnaire, the Fear of Progression Questionnaire-Short Form (FoP-Q-SF), the Multidimensional Scale of Perceived Social Support (MSPSS), and the Post-Traumatic Growth Inventory (PTGI). A total of 225 questionnaires were distributed, yielding 216 valid responses. Statistical analyses were performed using R software (version 4.5.2). The psych package was used for descriptive statistics, Pearson correlation analysis, and Harman’s single-factor test to assess common method bias. Structural equation modelling with maximum likelihood estimation was performed using the lavaan package. The indirect effect of PSS was tested using percentile bootstrap confidence intervals (5,000 resamples).

**Results:**

The mean PTG score was 41.26 ± 16.41. Pearson correlation analysis revealed a negative association between FoP and PTG (r = −0.454, *P* < 0.01), a negative association between FoP and PSS (r = −0.335, *P* < 0.01), and a positive association between PSS and PTG (r = 0.387, *P* < 0.01). Mediation analysis indicated that PSS accounted for a significant indirect effect in the association between FoP and PTG, representing 19.4% of the total effect.

**Conclusion:**

An indirect association between FoP and PTG via PSS was identified in this study. These findings suggest that social support and reduced fear of disease progression may be associated with greater psychological growth among lung cancer patients undergoing chemotherapy. Social support may warrant consideration as a potential intervention target for fostering positive psychological adaptation.

## 1 Introduction

According to data from the International Agency for Research on Cancer (IARC) in 2022, lung cancer remains the most prevalent cancer globally, with approximately 2.48 million new cases diagnosed annually worldwide. In China, around 1.06 million individuals are diagnosed with lung cancer each year, representing nearly 40% of the global incidence (1). Notably, the majority of Chinese lung cancer patients are diagnosed at intermediate or advanced stages, resulting in an overall five-year survival rate of less than 20% (2). Chemotherapy, a cornerstone of comprehensive lung cancer treatment, can extend survival but is also associated with adverse effects, including nausea, vomiting, hair loss, bone marrow suppression, and neurotoxicity (3–5). In line with the biopsychosocial model of cancer suffering, physical symptoms can influence psychosocial distress through patients’ cognitive appraisals of stimuli related to cancer diagnosis, treatment, and survival. These appraisals involve interpretations, judgements, and beliefs about cancer-related events or cues. Among these, fear of progression (FoP) represents a predominant factor (6). Research has identified FoP as one of the most common psychological distresses among lung cancer patients (7).

FoP is a reactive, conscious fear that arises in response to the reality of illness, its biological, psychological, and social consequences, and the potential for disease progression (8).This fear not only reflects patients’ understanding of their disease trajectory but also encompasses concerns about future quality of life, family responsibilities, and changes to personal identity (9). It has been shown to negatively impact patients’ quality of life (10, 11). Cross-sectional studies indicate that 37% to 78% of lung cancer patients experience moderate to severe FoP (12), and this fear can persist for up to 5 years or longer after diagnosis (13). Chronic excessive anxiety not only reduces treatment adherence and accelerates disease deterioration (14) but also leads to overmedicalization, thereby exacerbating the disease burden (15). Therefore, it is essential to address the FoP among lung cancer patients undergoing chemotherapy (16).

Post-Traumatic Growth (PTG) is a concept introduced by Tedeschi and Calhoun (17, 18), describing the positive psychological, emotional, and social changes that individuals undergo following major traumatic events (such as illness, disasters, or violence) through active coping and adaptation. PTG represents more than a return to a pre-trauma state; it reflects a transcendence of that state, involving self-improvement and enhanced functioning. In cancer research, studies have shown that PTG can promote the proliferation of white blood cells and lymphocytes, positively influencing immune function (19, 20). It may also alleviate psychological distress, enhance well-being, and improve quality of life (21, 22). However, from a psychological resource perspective, FoP may deplete individual psychological reserves, thereby limiting positive reflection and psychological transformation (23). A cross-sectional study among patients with oral cancer revealed a negative correlation between elevated FoP scores and PTG (24). Similarly, among patients with cervical cancer, FoP was negatively associated with PTG; this relationship was mediated by FoP’s undermining of patients’ self-care self-efficacy and health-promoting behaviors (25). Therefore, this study proposes Hypothesis 1: Among lung cancer patients undergoing chemotherapy, FoP will be negatively correlated with PTG.

Perceived social support (PSS) refers to the emotional experience and satisfaction that individuals derive from feeling supported, respected, and understood across various social domains. Specifically, it encompasses the support that individuals perceive from family, friends, and other social sources, emphasizing the subjective perception and interpretation of social support (26, 27). The main effects model of social support posits that social support has a universal beneficial impact on both physiological and psychological health, regardless of the level of support an individual receives (28). Additionally, the buffering model suggests that social support indirectly promotes health by mitigating the negative impact of stressful events and by directly reducing individuals’ perceived fear of illness (29). Empirically, Zheng et al. found that social support was associated with reduced FoP among post-operative lung cancer patients (30). Furthermore, Zhong et al. found that individuals with higher levels of PSS are more likely to seek assistance from medical professionals or close friends and family when confronting illness challenges. Seeking such assistance alleviates negative emotions and enhances confidence in overcoming the disease, thereby effectively reducing FoP levels (31). Additionally, research generally indicates that higher PSS predicts higher PTG (32–35). Jiao’s research suggests that in response to stressful situations (such as a cancer diagnosis), PSS functions as a protective external resource, facilitating patients’ positive reflection on traumatic events and thereby promoting PTG (36). Similarly, Gao and Shi’s study among breast cancer patients identified PSS as a key predictor of individual PTG (37).

The life crisis and personal growth model offers an integrative framework for understanding the aforementioned relationships (38). This model emphasizes that in coping with negative life events, individuals must achieve psychological growth through cognitive, emotional, and behavioral adjustments, supported by external resources such as perceived social support (38). Integrating these findings suggests that PSS is associated with lower FoP and may be associated with positive psychological transformation, potentially serving as a psychosocial pathway linking FoP and PTG. Therefore, this study proposes Hypothesis 2: Among lung cancer patients undergoing chemotherapy, PSS may mediate the relationship between FoP and PTG.

**Figure 1:**
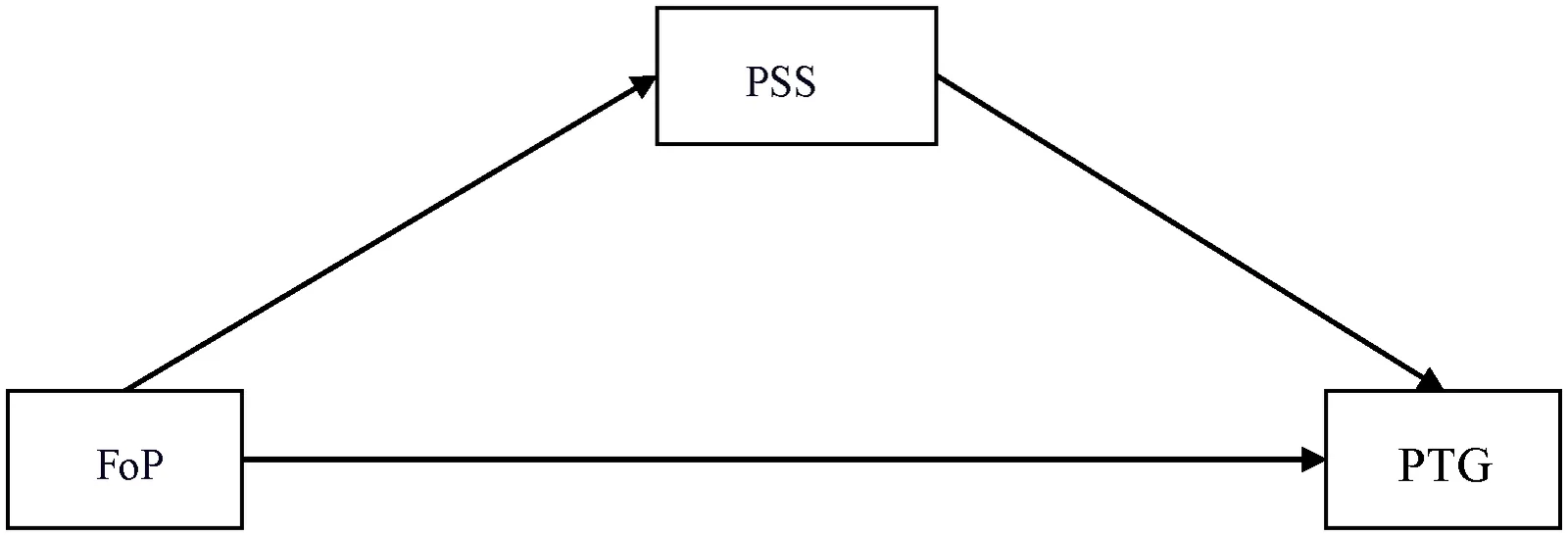
Hypothesized theoretical model. FoP, fear of Progression; PSS, Perceived Social Support ;PTG, Post-Traumatic Growth.

Currently, research on the relationship between FoP and PTG has predominantly focused on patients with oral, cervical, and breast cancer (24, 25, 39), with limited attention to lung cancer patients undergoing chemotherapy. Consequently, this study adopts the life crisis and personal growth model as its theoretical framework (38), conceptualizing FoP as a significant life crisis and PSS as a vital external resource. Specifically, this model positions external resources as a transmission mechanism between crisis events and growth outcomes. These resources buffer the psychological impact of adverse experiences and facilitate cognitive restructuring, which may transform crisis into growth opportunities. Within this framework, PSS may function as a psychosocial pathway linking FoP and PTG, potentially attenuating the negative implications of fear of disease progression while fostering positive psychological adaptation. The study hypotheses are formulated accordingly, with the conceptual model illustrated in **Figure 1**. The primary objectives are to: (1) examine the association between FoP and PTG among lung cancer patients undergoing chemotherapy; and (2) test whether PSS statistically mediates this association.

## 2 Materials and methods

### 2.1 Study design

A cross-sectional study was conducted among lung cancer patients receiving chemotherapy at a tertiary Grade A hospital in Deyang City, China, between March and July 2025. This study aimed to examine the association between fear of progression (FoP) and post-traumatic growth (PTG), and to test the indirect effect of perceived social support (PSS) in this relationship.

### 2.2 Participants

Participants were included in this study based on the following criteria: (1) Patients diagnosed with primary bronchogenic carcinoma via histopathological examination and admitted for chemotherapy; (2) No cognitive or communication impairments, with the capacity to understand the study content; (3) Age ≥ 18 years; (4) Expected survival ≥ 6 months; (5) Voluntary participation. Exclusion criteria included: (1) Patients scheduled for surgery following diagnosis; (2) Patients with organic brain disease or severe disease in other major organs (e.g., heart, liver, lungs); (3) Patients with concurrent malignancies or severe complications; (4) Patients experiencing intracranial metastasis during treatment or discontinuing therapy prematurely; (5) Patients receiving protective medical care, such as those unaware of their cancer diagnosis; (6) Patients unable to cooperate with the questionnaire for other reasons.

To determine the required sample size, G*Power software (Version 3.1) was employed based on a linear regression model (40). With a power set at 0.95 and an alpha level at 0.05, and assuming an effect size f² of 0.15 (indicating a moderate effect according to Cohen’s criteria) (41), 16 independent variables were specified. Based on these parameters, G*Power calculated a minimum required sample size of 204. Accounting for a 10% attrition rate, the estimated required sample size was 225 cases. A total of 225 questionnaires were distributed, yielding 216 valid responses, with a response rate of 96.0% (42).

### 2.3 Ethics statement

Ethical approval for this study was obtained from the Ethics Committee of Deyang People’s Hospital (approval number: 202404077K01). Prior to the survey, trained investigators explained the purpose, content, and completion guidelines of the study to the participants. Written informed consent was obtained from each participant before distributing the paper-based questionnaires for anonymous completion.

### 2.4 Data collection

Data were collected using paper-based questionnaires. Eligible participants completed a general information form, the Fear of Progression Questionnaire-Short Form (FoP-Q-SF), the Multidimensional Scale of Perceived Social Support (MSPSS), and the Post-Traumatic Growth Inventory (PTGI). The instruments were self-administered; for patients with limited literacy or visual impairment, trained researchers provided face-to-face assistance by reading each item aloud and documenting responses verbatim. Disease-related variables (e.g., cancer stage and comorbidities) were extracted from electronic medical records or confirmed by the attending clinicians, with patient consent.

### 2.5 Measures

#### 2.5.1 Demographic characteristics and disease-related information

This study utilized a general information questionnaire to gather participants’ demographic details, which included age, gender, number of children, marital status, educational level, religious affiliation, employment status, monthly household income per capita, place of residence, smoking history, drinking history, cancer clinical stage and number of comorbid chronic conditions. The categorization of all these variables is detailed in **Table 1**.

**Table 1:** Demographic and disease-related characteristics of lung cancer patients undergoing chemotherapy.

Variable	Category	N(%)	PTG(Mean ± SD)	*t\F*	*P*	LSD
Gender	Male	120(55.6)	41.88±17.50	0.630^*^	0.529	
	Female	96(44.4)	40.49±14.98			
Age(year)	18~44	13(6.0)	49.08±14.96	1.635	0.197	
	*45~59*	79(36.6)	41.25±15.78			
	*≥60*	124(57.4)	40.45±16.84			
Number of children	0	12(5.6)	41.75±15.31	0.211	0.889	
	1	138(63.9)	41.41±16.46			
	2	56(25.9)	40.21±17.02			
	≥3	10(4.6)	44.50±15.22			
Marital status	Unmarried	9(4.2)	44.00±13.92	0.247	0.782	
	Married	151(69.9)	40.81±16.80			
	Divorced/Widowed	56(25.9)	42.05±15.88			
Educational level	Primary school and below^a^	63(29.2)	36.25±16.93	4.318	0.015	b,c>a
	Secondary school^b^	101(46.8)	43.05±16.71			
	College and above^c^	52(24.1)	43.87±13.95			
Religious affiliation	No	177(81.9)	41.55±16.46	0.544^*^	0.589	
	Yes	39(18.1)	39.97±16.33			
Employment status	Employment^a^	95(44.0)	39.61±17.70	3.177	0.044	b>a,c
	Retirement^b^	42(19.4)	46.90±13.69			
	Unemployed^c^	79(36.6)	40.25±15.64			
Household monthly income per capita(Yuan)	<3000^a^	42(19.4)	36.86±16.68	3.679	0.013	d>a,b
	3000-4999^b^	97(44.9)	40.36±15.56			c>a
	5000-9999^c^	60(27.8)	42.88±16.96			
	≥10000^d^	17(7.9)	51.59±14.57			
Place of residence	Urban	62(28.7)	41.23±17.02	0.301	0.740	
	Township	64(29.6)	42.50±17.22			
	Rural	90(41.7)	40.41±15.50			
Smoking history	No	58(26.9)	41.09±15.41*	-0.100^*^	0.920	
	Yes	158(73.1)	41.33±16.81			
Drinking history	No	56(25.9)	40.82±15.70*	-0.241^*^	0.810	
	Yes	160(74.1)	41.42±16.69			
Cancer clinical stage	I	35(16.2)	44.26±15.36	1.031	0.380	
	II	80(37.0)	42.41±15.58			
	III	76(35.2)	39.63±17.66			
	IV	25(11.6)	38.36±16.40			
Number of comorbid chronic conditions	0 ^a^	49(22.7)	43.92±17.76	3.945	0.021	c<a,b
	1~2 ^b^	90(41.7)	43.37±16.60			
	≥3 ^c^	77(35.6)	37.12±14.58			

^*^ t-test. PTG, Post-Traumatic Growth. a, b, c denote different groups.

#### 2.5.2 Fear of progression questionnaire-short form

This scale was simplified by Mehnert et al. in 2006 based on the Fear of Progression Questionnaire (FoR-Q) (8), with a Cronbach’s α coefficient of 0.87. Wu Qiyun et al. adapted it into Chinese for use in China (43). It comprises two dimensions—Physical Health and Social Family—with a total of 12 items. Responses are scored on a five-point Likert scale ranging from ‘never’ (1 point) to ‘always’ (5 points), yielding a total score from 12 to 60 points. Higher scores indicate greater patient fear of disease progression. In this study, the Cronbach’s α coefficient for this scale was 0.814.

#### 2.5.3 Multidimensional scale of perceived social support

This scale was developed by Zimet (26) and subsequently adapted into Chinese by Jiang Qianjin (44). This scale consists of three dimensions: Family Support, Friend Support, and Other Support, comprising a total of 12 items. Responses are scored on a 7-point Likert scale, ranging from ‘Strongly Disagree’ (1 point) to ‘Strongly Agree’ (7 points). The total score ranges from 12 to 84, with higher scores indicating greater PSS. In this study, the Cronbach’s alpha coefficient for this scale was 0.950.

#### 2.5.4 Post-traumatic growth inventory

This scale was developed by Tedeschi and Calhoun in 1996 (17) and translated into Chinese by Wang in 2011 (45). The Cronbach’s α coefficient for the overall scale is 0.874, with the Cronbach’s α coefficients of its dimensions ranging from 0.611 to 0.796. This instrument primarily assesses positive changes experienced by individuals following a traumatic event. It comprises 20 items across five dimensions: appreciation of life, personal strength, new possibilities, relating to others, and spiritual change. Responses are scored on a 6-point Likert scale, ranging from 0 (“I did not experience this change as a result of my crisis”) to 5 (“a very great degree”). The total score ranges from 0 to 100, with higher scores indicating a higher level of PTG. In this study, the Cronbach’s α coefficient for the PTGI was 0.956.

### 2.6 Data analysis

Data were analyzed using R software (version 4.5.2). Continuous variables were described as mean ± standard deviation, and categorical variables were described as frequency and proportion. Differences in PTG scores between groups were examined using t-tests and one-way analysis of variance, with least significant difference *post-hoc* comparisons. Pearson correlation analysis was used to examine the correlations among FoP, PSS, and PTG. Missing data were handled using listwise deletion, and cases with missing values on any variable included in the model were excluded. Outliers were screened using Mahalanobis distance based on core scale items, with *P* < 0.001 as the critical value, and no extreme outliers requiring removal were identified. Common method bias was assessed using Harman’s single-factor test. Structural equation modeling was conducted using the lavaan package to construct a mediation model with FoP as the independent variable, PSS as the mediator, and PTG as the dependent variable. To control for confounding effects on path estimates, we included the following variables as covariates: variables showing statistically significant differences in PTG scores in univariate analysis (educational level, occupational status, household monthly income per capita, and number of comorbid chronic conditions), demographic characteristics (gender and age), and disease-related characteristics (cancer clinical stage). Specifically, gender was coded as a binary variable (female = 0, male = 1). Age was treated as a continuous variable and standardized. Educational level, household monthly income per capita, number of comorbid chronic conditions, and cancer clinical stage were treated as ordinal categorical variables and entered as numeric values. Occupational status was treated as a nominal categorical variable, with ‘employed’ as the reference group; two dummy variables were created for ‘retired’ and ‘unemployed’. Model parameters were estimated using the maximum likelihood method, and the percentile bootstrap method (5000 resamples) was used to calculate 95% confidence intervals for effect estimates to test the significance of the indirect effect (46). The significance level was set at 0.05.

## 3 Results

### 3.1 Demographic characteristics and disease-related information

A total of 216 patients were included in this study, with a mean age of 62.93 years (SD = 11.64; range 37–83). Of these patients, 55.6% were male, 57.4% were aged 60 years or older, 94.4% had children, 69.9% were married, 75.9% had an educational level below college, 81.9% had no religious affiliation, 36.6% were unemployed, 64.4% had a monthly household income per capita below ¥5,000, 41.7% lived in rural areas, 73.1% had a history of smoking, 74.1% had a history of drinking, 46.8% had stage III–IV cancer, and 77.3% had comorbid chronic diseases. Details are presented in **Table 1**.

Further comparisons of PTG scores among lung cancer patients undergoing chemotherapy revealed significant differences based on educational level, employment status, household monthly income per capita, and the number of comorbid chronic conditions. Specifically, patients with secondary school education or higher had significantly higher PTG scores compared to those with primary school education or below (*P* < 0.05). Retired patients exhibited significantly higher PTG scores than those who were employed or unemployed (*P* < 0.05). In terms of household monthly income per capita, patients with incomes exceeding ¥10,000 had significantly higher PTG scores than those with incomes below ¥3,000 or between ¥3,000 and ¥4,999 (*P* < 0.05). Additionally, patients with incomes between ¥5,000 and ¥9,999 had significantly higher PTG scores than those with incomes below ¥3,000 (*P* < 0.05). Furthermore, patients with three or more comorbid chronic conditions had significantly lower PTG scores compared to those with one or two chronic conditions or no additional chronic conditions (*P* < 0.05). For detailed results, refer to **Table 1**.

### 3.2 Statistical description of variables and Pearson correlation analysis

As shown in **Table 2**, the total FoP score of lung cancer patients undergoing chemotherapy was 40.66 ± 6.21, the total PSS score was 53.52 ± 15.48, and the total PTG score was 41.26 ± 16.41, suggesting that the PTG of this population was at a moderately low level. Pearson correlation analysis revealed that FoP was negatively correlated with PTG (r = -0.454, *P* < 0.01), FoP was negatively correlated with PSS (r = -0.335, *P* < 0.01), and PSS was positively correlated with PTG (r = 0.387, *P* < 0.01). As shown in **Table 3**.

**Table 2:** FoP, PSS, PTG and dimension scores for lung cancer patients undergoing chemotherapy(n=216).

Variable	MIN	MAX	M±SD
Physical Health	11	28	20.36±3.55
Social Family	11	29	20.30±3.28
FoP	22	53	40.66±6.21
Family Support	4	28	17.63±5.54
Friend Support	4	28	17.37±5.50
Other Support	4	28	18.53±5.70
PSS	16	84	53.52±15.48
New Possibilities	1	27	12.23±5.16
Personal Strength	0	14	6.14±2.84
Spiritual Change	0	18	8.27±3.83
Relating to Others	0	13	6.18±2.59
Appreciation of Life	0	17	8.44±3.30
PTG	4	85	41.26±16.41

FoP, fear of Progression; PSS, Perceived Social Support; PTG, Post-Traumatic Growth.

**Table 3:** Correlation between FoP, PSS and PTG in lung cancer patients undergoing chemotherapy (n=216).

Variable	1	2	3	4	5	6	7	8	9	10	11	12	13
1.Physical Health	1												
2.Social Family	0.657^**^	1											
3.FoP	0.917^**^	0.903^**^	1										
4.Family Support	-0.363^**^	-0.281^**^	-0.355^**^	1									
5.Friend Support	-0.337^**^	-0.242^**^	-0.320^**^	0.802^**^	1								
6.Other Support	-0.262^**^	-0.205^**^	-0.258^**^	0.729^**^	0.825^**^	1							
7.PSS	-0.346^**^	-0.262^**^	-0.335^**^	0.910^**^	0.945^**^	0.921^**^	1						
8.New Possibilities	-0.448^**^	-0.363^**^	-0.448^**^	0.360^**^	0.334^**^	0.290^**^	0.354^**^	1					
9.Personal Strength	-0.458^**^	-0.407^**^	-0.476^**^	0.385^**^	0.359^**^	0.299^**^	0.375^**^	0.880^**^	1				
10.Spiritual Change	-0.351^**^	-0.324^**^	-0.371^**^	0.286^**^	0.297^**^	0.284^**^	0.312^**^	0.794^**^	0.778^**^	1			
11.Relating to Others	-0.415^**^	-0.344^**^	-0.418^**^	0.393^**^	0.360^**^	0.302^**^	0.380^**^	0.895^**^	0.881^**^	0.792^**^	1		
12.Appreciation of Life	-0.374^**^	-0.302^**^	-.373^**^	0.321^**^	0.334^**^	0.306^**^	0.346^**^	0.753^**^	0.726^**^	0.776^**^	0.827^**^	1	
13.PTG	-0.446^**^	-0.378^**^	-0.454^**^	0.386^**^	0.369^**^	0.321^**^	0.387^**^	0.936^**^	0.931^**^	0.882^**^	0.966^**^	0.883^**^	1

FoP, fear of Progression; PSS, Perceived Social Support; PTG, Post-Traumatic Growth. **P < 0.01.

### 3.3 Analysis of the indirect effect of PSS between FoP and PTG in lung cancer patients undergoing chemotherapy

Harman’s single-factor test was employed to conduct exploratory factor analysis on all items related to FoP, PSS, and PTG included in this study. A total of eight common factors with eigenvalues exceeding 1 were identified. The first common factor accounted for 33.11% of the variance, which is below the critical threshold of 40% (39). To further assess common method bias, we compared the fit of a three-factor model with that of a single-factor model using confirmatory factor analysis (CFA). In the three-factor model, FoP, PSS, and PTG were specified as distinct latent variables; in the single-factor model, all items were constrained to load on a common factor. The three-factor model yielded χ² = 2212.1 (df = 899), CFI = 0.794, TLI = 0.783, RMSEA = 0.082, and SRMR = 0.068, whereas the single-factor model yielded χ² = 4060.2 (df = 902), CFI = 0.505, TLI = 0.480, RMSEA = 0.127, and SRMR = 0.141. The substantially better fit of the three-factor model relative to the single-factor model indicates adequate discriminant validity among FoP, PSS, and PTG, suggesting that common method bias did not severely compromise the study findings.

**Figure 2:**
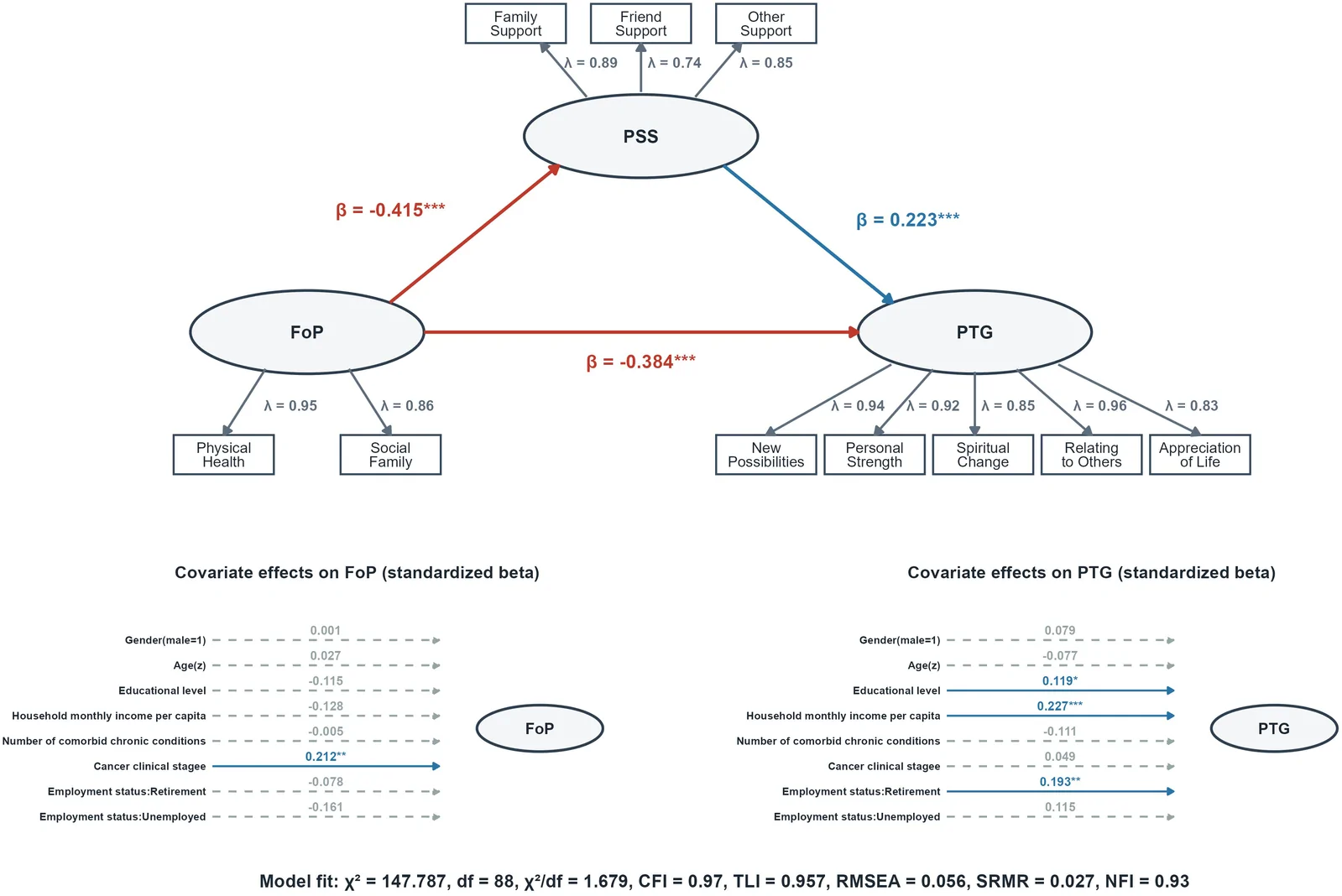
Covariate-adjusted structural equation model of the relationships among FoP, PSS and PTG. Ellipses represent latent variables and rectangles represent observed indicators. Values on arrows from latent variables to indicators are standardized factor loadings (λ); values on arrows between latent variables are standardized path coefficients (β). FoP, fear of Progression; PSS, Perceived Social Support ;PTG, Post-Traumatic Growth. *P<0.05, **P<0.01, **P<0.001.

The structural equation model was further constructed using the lavaan package in R (**Figure 2**). In this model, FoP was designated as the independent variable, PSS as the intermediary variable, and PTG as the dependent variable. The model was estimated and tested using maximum likelihood estimation. The results indicated a good model fit: χ² = 147.787, df = 88, χ²/df=1.679, RMSEA = 0.056 (90% CI: 0.040, 0.072), SRMR = 0.027, IFI = 0.970, CFI = 0.970, NFI = 0.930, TLI = 0.957.

The Bootstrap procedure (with 5,000 resamples) was employed to conduct significance testing and calculate the 95% confidence intervals (95% CIs) for effect sizes, further decomposing and verifying the direct and indirect effects between variables through path influences. The results indicated that the direct effect of FoP on PTG was -0.384, the direct effect of FoP on PSS was -0.415, the direct effect of PSS on PTG was 0.223, the indirect effect of PSS between FoP and PTG was -0.092, and the total effect of FoP on PTG was -0.477. The 95% confidence intervals for all effects excluded zero, with the indirect effect accounting for 19.4% of the total effect. Detailed results are presented in **Table 4** and **Figure 2**.

**Table 4:** Mediating effects of PSS between FoP and PTG in lung cancer patients undergoing chemotherapy.

Path	Effect	BootSE	*95%CI*	Ratio of effect
Total path of FoP on PTG	-0.477	0.071	-0.552 ~ -0.268	–
Direct path of FoP on PTG	-0.384	0.077	-0.484 ~ -0.179	80.6%
Indirect path of FoP on PTG	-0.092	0.033	-0.157 ~ -0.029	19.4%

FoP, fear of Progression; PSS, Perceived Social Support; PTG, Post-Traumatic Growth.

## 4 Discussion

In recent years, the incidence of lung cancer has increased steadily due to risk factors such as smoking, air pollution, and occupational exposure, posing a significant global public health challenge. Lung cancer patients undergoing chemotherapy often face substantial difficulties in disease management, psychological adaptation, and trauma processing, all of which may severely impact their quality of life and recovery outcomes. Research indicates that enhancing PTG may be associated with psychological transformation, enabling patients to better manage their illness and improve prognosis (47, 48). This study employs the life crisis and personal growth model as its theoretical framework to investigate whether PSS mediates the relationship between FoP and PTG in lung cancer patients undergoing chemotherapy. It aims to elucidate the mechanism by which PSS contributes to psychological rehabilitation, providing a robust theoretical basis for developing precise and effective psychosocial support strategies for cancer patients.

In this study, the mean PTG score was 41.26 ± 16.41. This is significantly lower than the score reported by Zhang et al. (49) for Chinese cancer survivors (61.15 ± 20.26) and also lower than that reported by Peng et al. (50) for lung cancer patients (45.52 ± 11.17). This suggests suboptimal psychological growth among lung cancer patients undergoing chemotherapy. The poor prognosis and severe symptoms of lung cancer may induce intense illness perception, potentially consuming substantial psychological resources (51). This may make it difficult for patients to redirect their attention toward positive psychological transformation, potentially limiting PTG. Concurrently, chemotherapy frequently entails side effects such as nausea, vomiting, hair loss, and immunosuppression. These side effects may exacerbate physiological burdens and provoke negative emotions such as inferiority and anxiety, potentially reducing psychological adaptive capacity (52, 53) and PTG levels. Moreover, within East Asian cultural contexts, cancer patients may be inclined to conceal their emotions and avoid seeking psychological support because of disease-related stigma (54). This may limit their ability to confront and adjust their psychological state, potentially affecting PTG. These factors may explain why PTG scores in this study were lower than those reported in other research.

Demographic characteristics revealed significant differences in patients’ PTG scores across educational levels, employment status, household monthly income per capita, and number of comorbid chronic conditions. Compared to patients with primary education and below, those with secondary education, college education and above demonstrated higher PTG scores (*P* < 0.05). Wang’s research similarly indicates a positive correlation between educational attainment and PTG (55). This may possibly reflect that higher-educated individuals possess greater cognitive capacity and analytical skills, which may enable a deeper understanding of traumatic events and the potential for growth. Furthermore, a higher education background may imply access to broader social resources and support systems, potentially providing individuals with more coping resources. In this study, retired patients demonstrated significantly higher PTG scores than employed and unemployed patients (*P* < 0.05), mirroring findings from Wang et al.’s investigation of Chinese breast cancer patients (56). This disparity might be explained by the possibility that retired individuals have greater discretionary time to manage illness and treatment, as well as to foster family relationships.

Patients with a household monthly income per capita exceeding ¥10,000 demonstrated significantly higher PTG scores than those with incomes below ¥3,000 or between ¥3,000 and ¥4,999 (P < 0.05). Similarly, patients with household monthly income per capita between ¥5,000 and ¥9,999 exhibited significantly higher PTG scores than those below ¥3,000 (P < 0.05), consistent with findings reported by Cui et al. (57). The protracted duration and substantial cost of lung cancer chemotherapy impose a heavy burden on patients (58). Patients with higher household incomes may experience relatively less financial pressure, enabling them to devote more time and energy to treatment and recovery (59), and may be more likely to adopt proactive psychological adjustment strategies, potentially associated with greater PTG. Furthermore, patients with ≥3 concurrent chronic conditions exhibited significantly lower PTG scores than those with 1–2 chronic conditions or no additional chronic diseases (P < 0.05). This could potentially be explained by the possibility that coexisting chronic illnesses, alongside lung cancer, may exacerbate physical burdens, induce various complications, and increase treatment costs, thereby imposing substantial psychological and financial strain on patients (60), which may contribute to lower PTG levels.

However, this study did not find significant associations for certain factors previously linked to PTG. For instance, research indicates a significant correlation between religious beliefs and PTG (61). Some studies have reported a positive correlation between age and PTG (55), whereas others have reported a negative correlation (62). These discrepancies may be related to differing research methodologies, sample characteristics, and other potential confounding factors, warranting further exploration in future studies.

As hypothesized, this study found a significant negative correlation between FoP and PTG in lung cancer chemotherapy patients, consistent with Hypothesis 1. Individuals with lower FoP levels tended to report higher PTG, consistent with Du’s findings (63). According to Hirsch and Mathews’ illness worry model, high FoP may induce excessive vigilance and intrusive thoughts, potentially leading patients to fixate on negative threats (64). This may divert substantial psychological resources towards managing anxiety about disease progression, potentially limiting engagement in the positive regulatory processes necessary for psychological growth (65) and potentially limiting PTG development. Conversely, individuals with lower FoP levels may more readily shift attention from negative impacts to positive changes, which is associated with higher PTG. Furthermore, Tedeschi and Calhoun’s PTG model posits that PTG requires individuals to undergo cognitive restructuring (18). However, excessive preoccupation with disease deterioration among lung cancer chemotherapy patients may trigger intense negative emotional responses (66), depleting cognitive resources and hindering effective cognitive restructuring (67). This may limit positive growth following traumatic experiences. Therefore, we recommend that healthcare providers prioritize disease comprehension and fear assessment, offering timely support to patients with heightened negative disease cognition. Proactive intervention may help patients mitigate fears regarding their illness and potential complications, achieve psychological growth, and confront disease and treatment regimens more effectively. Ultimately, this may enhance overall quality of life and treatment adherence, contributing to improved long-term health outcomes.

This study employed structural equation modelling to examine whether PSS was statistically associated with an indirect effect in the association between FoP and PTG after controlling for gender, age, education, occupation, income, comorbid chronic conditions, and cancer stage. These findings were consistent with Hypothesis 2: higher FoP was associated with lower levels of PSS and reduced PTG; conversely, lower FoP was associated with higher PSS and greater PTG. The mediation model was statistically significant, suggesting that PSS accounted for a significant indirect effect in the relationship between FoP and PTG. This indirect effect model further supports the applicability of the life crisis and personal growth model among lung cancer patients undergoing chemotherapy, providing empirical evidence for this theoretical framework. From a cognitive-behavioral perspective (68), PSS may be associated with PTG by altering individuals’ cognitive appraisals of traumatic events, enabling them to interpret their experiences from a more positive standpoint; this cognitive restructuring process is positively associated with PTG. Additionally, elevated FoP may direct excessive attention toward health concerns, making patients prone to overlooking available support. This tendency toward social withdrawal is associated with lower levels of PSS (69, 70). According to social support theory, social support provides emotional comfort, informational exchange, and practical assistance that help individuals cope with stress and challenges, which may contribute to positive psychological transformation (28, 29). Therefore, FoP may be indirectly associated with lower PTG through diminished PSS.

The results of this study are consistent with an indirect effect of PSS between FoP and PTG. Moreover, the life crisis and personal growth model emphasizes the supportive role of external resources in psychological growth. In this study, PSS, as a key external resource, showed a significant indirect effect even after controlling for multiple covariates, further supporting the applicability of this model among lung cancer patients undergoing chemotherapy. Future work may prioritize social support as a focal point for interventions, optimizing therapeutic outcomes by encouraging patients to actively seek beneficial social networks, strengthening emotional companionship and support within families, and fostering trust and understanding within the doctor-patient relationship. Given that patients’ needs for social support vary, interventions should be tailored to specific patient requirements. For instance, research indicates that anxious individuals may require more companionship support, whereas younger patients may rely more heavily on family support (71). This approach may not only enhance the effectiveness of social support resources but also improve their utility in alleviating patient fears. Furthermore, as primary hubs for social support, hospitals should fully leverage digital technologies to establish online platforms offering professional guidance, which may support patients’ positive psychological transformation and growth.

## 5 Conclusion

Our findings support the relationship between FoP and PTG. They highlight the indirect association of PSS between FoP and PTG. This effect has been insufficiently explored among lung cancer chemotherapy patients. Given that PSS is recognized as a key psychological factor, interventions targeting social support may be beneficial for patients. This may support patients’ attainment of positive psychological transformation, which may improve their overall quality of life.

## 6 Limitations

This study also has certain limitations. Firstly, the cross-sectional design precludes establishing causal relationships between FoP, PSS, and PTG. Future longitudinal or randomized controlled trials would be needed to validate these findings. Secondly, the non-random sampling method may have introduced selection bias during recruitment, potentially compromising the representativeness of the sample. This necessitates caution when interpreting results. Thirdly, data were collected through a combination of self-administered questionnaires and researcher-assisted oral administration, in which researchers read the items aloud and recorded patients’ responses verbatim. The concurrent use of these two modes may have introduced measurement bias due to social desirability effects and inconsistencies in data collection. Future studies should adopt a uniform mode of administration to minimize measurement error. Additionally, the indirect effect of PSS accounted for only 19.4% of the total effect between FoP and PTG, leaving approximately 80% of this relationship unexplained. Other psychosocial variables, such as resilience, coping style, illness uncertainty, self-efficacy, depression, and family functioning, may play equally or more important roles in mediating or moderating the relationship between FoP and PTG. Finally, owing to practical constraints, the study sample was limited to lung cancer chemotherapy patients from a single tertiary hospital in Deyang, a medium-sized city in southwestern China. Cultural norms in China emphasize familial collectivism and interdependence, which may inflate perceived social support scores compared with more individualistic societies. Healthcare access and insurance coverage patterns in this region may also differ from other provinces or countries. These factors may limit the generalizability of our findings, and caution is warranted when applying them to other regions or populations. Subsequent research should employ larger, multi-site samples to enhance external validity. Despite these limitations, this study contributes to the understanding of the mechanisms underlying FoP and PTG by integrating the life crisis and personal growth model.

## Data Availability

The raw data supporting the conclusions of this article will be made available by the authors, without undue reservation.

## References

[1] Bray F Laversanne M Sung H Ferlay J Siegel RL Soerjomataram I . Global cancer statistics 2022: Globocan estimates of incidence and mortality worldwide for 36 cancers in 185 countries. CA: A Cancer J For Clin. (2024) 74:229–63. doi: 10.3322/caac.21834 38572751

[2] Office of the National Health Commission . Guidelines for the diagnosis and treatment of primary lung cancer (2022 edition). Acta Med Sin. (2022) 13:549–70. doi: 10.12290/xhyxzz.2022-0352

[3] von Itzstein MS Rashdan S Dahlberg SE Gerber DE Sandler AB Schiller JH . Incidence and correlates of high-grade chemotherapy-induced peripheral neuropathy in patients with lung cancer. Oncologist. (2025) 30:oyaf036. doi: 10.1093/oncolo/oyaf036 40152312 PMC11950918

[4] Bai L Ni L Lu J Zhang YY Yin Y Zhang W . Relationship between nausea and vomiting and physical activity in patients with lung cancer undergoing first chemotherapy. Front Oncol. (2024) 14:1396637. doi: 10.3389/fonc.2024.1396637 39114312 PMC11303201

[5] Yu J Huang T Xu J Xiao J Chen Q Zhang L . Effect of nursing method of psychological intervention combined with health education on lung cancer patients undergoing chemotherapy. J Healthcare Eng. (2022) 2022:2438612. doi: 10.1155/2022/2438612 35222879 PMC8866018

[6] Hall DL Lennes IT Pirl WF Friedman ER Park ER . Fear of recurrence or progression as a link between somatic symptoms and perceived stress among cancer survivors. Supp Care Cancer. (2017) 25:1401–7. doi: 10.1007/s00520-016-3533-3 27966025 PMC5500975

[7] Zhang L Deng J Li Z Wu F Luo T Li F . Social support and self-efficacy mediate the link between health literacy and fear of disease progression in Chinese lung cancer patients. Sci Rep. (2026). doi: 10.1038/s41598-026-56265-3 42373682 PMC13518780

[8] Mehnert A Herschbach P Berg P Henrich G Koch U . Fear of progression in breast cancer patients--validation of the short form of the fear of progression questionnaire (Fop-Q-Sf). Z fur Psychosomatische Med und Psychotherapie. (2006) 52:274–88. doi: 10.13109/zptm.2006.52.3.274 17156600

[9] Lu Q Liu Q Fang S Ma Y Zhang B Li H . Relationship between fear of progression and symptom burden, disease factors and social/family factors in patients with stage-iv breast cancer in Shandong, China. Cancer Med. (2024) 13:e6749. doi: 10.1002/cam4.6749 38457242 PMC10923048

[10] Folkerts AK Haarmann L Nielsen J Saliger J Eschweiler M Karbe H . Fear of progression is determined by anxiety and self-efficacy but not disease-specific parameters in patients with Parkinson's disease: Preliminary data from a multicenter cross-sectional study. J Parkinson's Dis. (2022) 12:2543–53. doi: 10.3233/jpd-223314 36189603

[11] Deng H Yang T Hu Y Liu J Chou H Jiang Y . Symptom clusters, fear of disease progression, and quality of life in postoperative gastric cancer patients: A cross-sectional study. Supp Care Cancer. (2025) 33:219. doi: 10.1007/s00520-025-09180-8 39998660

[12] Chen R Yang H Zhang H Chen J Liu S Wei L . Fear of progression among postoperative patients with newly diagnosed lung cancer: A cross-sectional survey in China. BMC Psychol. (2023) 11:168. doi: 10.1186/s40359-023-01211-5 37217966 PMC10201766

[13] Herschbach P Dinkel A . Fear of progression. Recent Results Cancer Res Fortschr der Krebsforschung Progres dans les Recherches sur le Cancer. (2014) 197:11–29. doi: 10.1007/978-3-642-40187-9_2 24305766

[14] Endeshaw D Biresaw H Asefa T Yesuf NN Yohannes S . Sleep quality and associated factors among adult cancer patients under treatment at oncology units in Amhara region, Ethiopia. Nat Sci Sleep. (2022) 14:1049–62. doi: 10.2147/nss.S356597 35673619 PMC9167589

[15] Xiao Y Qing Feng W Di Fen L Wei Qi G . A study on the relationships among lung cancer survivors' fear of disease progression, anxiety about cancer death, and cancer self - efficacy. J Nurs Manage. (2022) 22:392–7. doi: 10.3969/j.issn.1671-315x.2022.06.003

[16] Tao XQ Wu QC Wei X Zhang W Zhou Y Qiu H . Fear of progression and its associated factors among patients with positive lung cancer screening results: A latent profile analysis. Nurs Health Sci. (2025) 27:e70176. doi: 10.1111/nhs.70176 40611604

[17] Tedeschi RG Calhoun LG . The posttraumatic growth inventory: Measuring the positive legacy of trauma. J Trauma Stress. (1996) 9:455–71. doi: 10.1007/BF02103658 8827649

[18] Dursun P Söylemez İ . Posttraumatic growth: A comprehensive evaluation of the recently revised model. Turk Psikiyatri Dergisi = Turkish J Psychiatry. (2020) 31:57–68. doi: 10.5080/u23694 32594480

[19] McGregor BA Antoni MH Boyers A Alferi SM Blomberg BB Carver CS . Cognitive-behavioral stress management increases benefit finding and immune function among women with early-stage breast cancer. J Psychosomatic Res. (2004) 56:1–8. doi: 10.1016/s0022-3999(03)00036-9 14987957

[20] Dunigan JT Carr BI Steel JL . Posttraumatic growth, immunity and survival in patients with hepatoma. Digestive Dis Sci. (2007) 52:2452–9. doi: 10.1007/s10620-006-9477-6 17417728

[21] Husson O Zebrack B Block R Embry L Aguilar C Hayes-Lattin B . Posttraumatic growth and well-being among adolescents and young adults (Ayas) with cancer: A longitudinal study. Supp Care Cancer. (2017) 25:2881–90. doi: 10.1007/s00520-017-3707-7 28424888 PMC5527055

[22] Onyedibe MC Blickle P Schmidt ME Steindorf K . Posttraumatic growth and health-related quality of life in cancer survivors: Does fatigue moderate the link? Stress Health: J Int Soc For Invest Stress. (2024) 40:e3299. doi: 10.1002/smi.3299 37547957

[23] Jiang Y Li H Xiong Y Zheng X Liu Y Zhou J . Association between fear of cancer recurrence and emotional distress in breast cancer: A latent profile and moderation analysis. Front Psychiatry. (2025) 16:1521555. doi: 10.3389/fpsyt.2025.1521555 40212837 PMC11983599

[24] Zhang X Yuan M Yue Y Duan X . Fear of cancer recurrence and coping strategies among patients with oral cancer: The impact on post-traumatic growth. BMC Psychol. (2025) 13:162. doi: 10.1186/s40359-025-02499-1 40001229 PMC11863465

[25] Li L Su Y . Mediation effects of self-care self-efficacy and health-promoting behaviors on fear of cancer recurrence and posttraumatic growth in postoperative patients with cervical cancer: A cross-sectional study. Asian Nurs Res. (2024) 18:468–78. doi: 10.1016/j.anr.2024.10.003 39427877

[26] Zimet G Dahlem N Zimet S Farley G . The multidimensional scale of perceived social support. J Pers Assess. (1988) 52:30–41. doi: 10.1207/s15327752jpa5201_2 2280326

[27] Sarason BR Pierce GR Shearin EN Sarason IG Waltz JA Poppe LD . Perceived social support and working models of self and actual others. J Pers Soc Psychol. (1991) 60:273–87. doi: 10.1037/0022-3514.60.2.273 27371692

[28] House JS Landis KR Umberson D . Social relationships and health. Sci (New York NY). (1988) 241:540–5. doi: 10.1126/science.3399889 3399889

[29] Cohen S Wills TA . Stress, social support, and the buffering hypothesis. psychol Bull. (1985) 98:310–57. doi: 10.1037/0033-2909.98.2.310 3901065

[30] Zheng W Hu M Liu Y . Social support can alleviate the fear of cancer recurrence in postoperative patients with lung carcinoma. Am J Trans Res. (2022) 14:4804–11. PMC9360850 35958474

[31] Zhong M She F Wang W Ding L Wang A . The mediating effects of resilience on perceived social support and fear of cancer recurrence in glioma patients. Psychol Res Behav Manage. (2022) 15:2027–33. doi: 10.2147/prbm.S374408 35967594 PMC9365061

[32] Chen M Che C . Perceived social support, self-management, perceived stress, and post-traumatic growth in older patients following stroke: Chain mediation analysis. Medicine. (2024) 103:e38836. doi: 10.1097/md.0000000000038836 39029078 PMC11398753

[33] Zahra K Khan S Sadia R Aslam I . Resilience and post-traumatic growth among cancer patients: A moderated mediation analysis through perceived social support and stress. Psychol Russia: State Art. (2024) 17:34–49. doi: 10.11621/pir.2024.0203 39552781 PMC11562008

[34] Xie CS Kim Y . Post-traumatic growth during Covid-19: The role of perceived social support, personality, and coping strategies. Healthcare (Basel). (2022) 10:224. doi: 10.3390/healthcare10020224 35206839 PMC8872023

[35] Şirin Gök M Çiftçi B . Relationship between perceived social support and post-traumatic growth in coronavirus disease 2019 patients discharged from the hospital. World J Psychiatry. (2023) 13:171–81. doi: 10.5498/wjp.v13.i4.171 37123100 PMC10130958

[36] Jie J Shuang L Zhiqin L Wanmin Q . A study on the correlation between post-traumatic growth and psychological resilience in primary carers of late-stage cancer patients and their social support. J Nurs Dev. (2020) 35:149–52. doi: 10.16821/j.cnki.hsjx.2020.02.014

[37] Ran G Tieying S . A study on the correlation between post-traumatic growth and general self-efficacy and perceived social support in breast cancer patients. Nurs Res. (2017) 31:281–4. doi: 10.3969/j.issn.1009-6493.2017.03.009

[38] Schaefer JA Moos RH . The context for posttraumatic growth: Life crises, individual and social resources, and coping. In: Posttraumatic Growth: Positive Changes in the Aftermath of Crisis. The Lea Series in Personality and Clinical Psychology. Lawrence Erlbaum Associates Publishers, Mahwah, NJ, US (1998). p. 99–125.

[39] Wen L Xiaohua S Susu H Jiaqian H Shanshan X Peng D . The mediating effect of fear of disease progression between post-traumatic stress disorder and post-traumatic growth in breast cancer patients. Military Nurs. (2025) 42:45–8. doi: 10.3969/j.issn.2097-1826.2025.07.012

[40] Faul F Erdfelder E Lang A-G Buchner A . G*Power 3: A flexible statistical power analysis program for the social, behavioral, and biomedical sciences. Behav Res Methods. (2007) 39:175–91. doi: 10.3758/BF03193146 17695343

[41] Cohen J . Chapter 4 - Differences between correlation coefficients. In: Cohen J , editor. Statistical Power Analysis for the Behavioral Sciences. Academic Press, New York (1977). p. 109–43.

[42] Kline RB . Principles and Practice of Structural Equation Modeling. 4th Ed. New York, NY, US: The Guilford Press (2016) p. xvii–534-xvii.

[43] Qiyun W Zhixia Y Li L Peiyu L . Chinese version and reliability and validity analysis of the shortened fear of progression questionnaire for cancer patients. Chin J Nurs. (2015) 50:1515–9. doi: 10.3761/j.issn.0254-1769.2015.12.021

[44] Qian Jin J . Comprehending the social support scale. Chin Sci Behav Med. (2001) 10:41–2.

[45] Ji W Chen Y Wang YB Liu XH . Revision and reliability analysis of the posttraumatic growth rating scale. J Nurs. (2011) 26:26–8. doi: 10.3870/hlxzz.2011.14.026

[46] Preacher KJ Hayes AF . Asymptotic and resampling strategies for assessing and comparing indirect effects in multiple mediator models. Behav Res Methods. (2008) 40:879–91. doi: 10.3758/brm.40.3.879 18697684

[47] Glazebrook AJ Shakespeare-Finch J Andrew B van der Meer J . Posttraumatic growth eeg neuromarkers: Translational neural comparisons with resilience and ptsd in trauma-exposed healthy adults. Eur J Psychotraumatol. (2023) 14:2272477. doi: 10.1080/20008066.2023.2272477 37965734 PMC10653763

[48] Liu N Zhang L Liu Y Ding X Li Q Lixia G . Relationship between self-psychological adjustment and post-traumatic growth in patients with lung cancer undergoing chemotherapy: A cross-sectional study. BMJ Open. (2024) 14:e081940. doi: 10.1136/bmjopen-2023-081940 38719309 PMC11086470

[49] Zhang L Lu Y Qin Y Xue J Chen Y . Post-traumatic growth and related factors among 1221 Chinese cancer survivors. Psychooncology. (2020) 29:413–22. doi: 10.1002/pon.5279 31705578

[50] Peng X Su Y Huang W Hu X . Status and factors related to posttraumatic growth in patients with lung cancer: A strobe-compliant article. Medicine. (2019) 98:e14314. doi: 10.1097/md.0000000000014314 30762731 PMC6408060

[51] Morrison EJ Novotny PJ Sloan JA Yang P Patten CA Ruddy KJ . Emotional problems, quality of life, and symptom burden in patients with lung cancer. Clin Lung Cancer. (2017) 18:497–503. doi: 10.1016/j.cllc.2017.02.008 28412094 PMC9062944

[52] Hesketh PJ Batchelor D Golant M Lyman GH Rhodes N Yardley D . Chemotherapy-induced alopecia: Psychosocial impact and therapeutic approaches. Supp Care Cancer. (2004) 12:543–9. doi: 10.1007/s00520-003-0562-5 15221580

[53] El Kheir DYM Ibrahim AHM . Epidemiological assessment of distress during chemotherapy: Who is affected? J Taibah Univ Med Sci. (2019) 14:448–53. doi: 10.1016/j.jtumed.2019.08.004 31728143 PMC6838912

[54] Wang Z Hu X Xu J Zhou J Ou X Chen M . Effectiveness of oh card-based group mental health education in improving mood and behavior in breast cancer patients. Front Public Health. (2025) 13:1533073. doi: 10.3389/fpubh.2025.1533073 40161016 PMC11949784

[55] Wang S Li N Li K Qiu Q Wu C Zhou R . Changes and influencing factors of post-traumatic growth in patients after enterostomy. Eur J Oncol Nurs. (2025) 76:102844. doi: 10.1016/j.ejon.2025.102844 40058271

[56] Wang ML Liu JE Wang HY Chen J Li YY . Posttraumatic growth and associated socio-demographic and clinical factors in Chinese breast cancer survivors. Eur J Oncol Nurs. (2014) 18:478–83. doi: 10.1016/j.ejon.2014.04.012 24958639

[57] Cui C Wang K An J Jin C . Current status and influencing factors of post-traumatic growth in maintenance hemodialysis. Int J Nurs Sci. (2017) 4:362–6. doi: 10.1016/j.ijnss.2017.09.008 31406778 PMC6626164

[58] Gourzoulidis G Kastanioti C Mavridoglou G Kotsilieris T Voudigaris D Tzanetakos C . Does real-world evidence of the economic burden of lung cancer in Greece exist? A systematic review of the literature. Curr Oncol (Toronto Ont). (2025) 32:130. doi: 10.3390/curroncol32030130 40136334 PMC11941143

[59] Yang K Hua S Wei W Yang C Zhu X Li SC . Economic burden of advanced lung cancer patients treated by gefitinib alone and combined with chemotherapy in two regions of China. J Med Econ. (2023) 26:1424–31. doi: 10.1080/13696998.2023.2272536 37855437

[60] Buja A Rugge M Di Pumpo M Zorzi M Rea F Pantaleo I . Healthcare costs by comorbidity patterns in lung cancer patients. Cancers. (2025) 17:2682. doi: 10.3390/cancers17162682 40867311 PMC12384978

[61] Kim YS Moon JH Lee YS Kim YW Heo GR Oh SK . Factors influencing posttraumatic growth in patients with lung cancer. J Korean Clin Nurs Res. (2021) 27:98–108. doi: 10.22650/JKCNR.2021.27.1.98

[62] Boyacıoğlu NE Temel M Çaynak S . Post-traumatic growth in cancer patients: A correlational study in Turkey. J Religion Health. (2022) 61:4366–81. doi: 10.1007/s10943-022-01574-w 35567645

[63] Du L Cai J Yu J Chen X Yang X Xu X . Relations between posttraumatic growth and fear of progression among young and middle-aged primary brain tumor patients: The parallel mediating role of perceived social support and illness uncertainty. World Neurosurg. (2024) 184:e794–802. doi: 10.1016/j.wneu.2024.02.048 38364895

[64] Hirsch CR Mathews A . A cognitive model of pathological worry. Behav Res Ther. (2012) 50:636–46. doi: 10.1016/j.brat.2012.06.007 22863541 PMC3444754

[65] Hobfoll SE . Conservation of resources theory: Its implication for stress, health, and resilience. In: The Oxford Handbook of Stress, Health, and Coping. Oxford Library of Psychology. Oxford University Press, New York, NY, US (2011). p. 127–47.

[66] Yang YL Zhang XQ Yang YQ Li EM Zhou B Gong YW . Relationship between uncertainty in illness and fear of progression among lung cancer patients: The chain mediation model. World J Psychiatry. (2025) 15:104979. doi: 10.5498/wjp.v15.i5.104979 40495845 PMC12147003

[67] Liu Y Wu W Li S Wang X Zhang L . Exploring psychological distress among lung cancer patients through the stress system model. Sci Rep. (2025) 15:27253. doi: 10.1038/s41598-025-09847-6 40715189 PMC12297220

[68] Beck AT . Cognitive Therapy and the Emotional Disorders. Oxford, England: International Universities Press (1976). p. 356.

[69] Hinz A Schulte T Mehnert-Theuerkauf A Richter D Sender A Brock H . Fear of cancer progression: A comparison between the fear of progression questionnaire (Fop-Q-12) and the concerns about recurrence questionnaire (Carq-4). Healthcare. (2024) 12:435. doi: 10.3390/healthcare12040435 38391810 PMC10888487

[70] Koch L Jansen L Brenner H Arndt V . Fear of recurrence and disease progression in long-term (≥ 5 years) cancer survivors--a systematic review of quantitative studies. Psychooncology. (2013) 22:1–11. doi: 10.1002/pon.3022 22232030

[71] Liu S Zhang Y Miao Q Zhang X Jiang X Chang T . The mediating role of self-perceived burden between social support and fear of progression in renal transplant recipients: A multicenter cross-sectional study. Psychol Res Behav Manage. (2023) 16:3623–33. doi: 10.2147/prbm.S424844 37693331 PMC10488562

